# Integral inequalities for some convex functions via generalized fractional integrals

**DOI:** 10.1186/s13660-018-1807-7

**Published:** 2018-08-14

**Authors:** Naila Mehreen, Matloob Anwar

**Affiliations:** School of Natural Sciences, National University of Sciences and Technology, Islamabad, Pakistan

**Keywords:** 26A51, 26A33, 26D10, 26D07, 26D15, Hermite–Hadamard inequalities, Riemann–Liouville fractional integral, Hadamard fractional integral, Katugampola fractional integral, Convex functions, *s*-convex functions, *m*-convex functions

## Abstract

In this paper, we obtain the Hermite–Hadamard type inequalities for *s*-convex functions and *m*-convex functions via a generalized fractional integral, known as Katugampola fractional integral, which is the generalization of Riemann–Liouville fractional integral and Hadamard fractional integral. We show that through the Katugampola fractional integral we can find a Hermite–Hadamard inequality via the Riemann–Liouville fractional integral.

## Introduction

A function $f:I\rightarrow \mathbb{R}$, where *I* is an interval of real numbers, is called convex if the following inequality holds:

1 $$ f\bigl(ta+(1-t)b\bigr)\leq tf(a)+(1-t)f(b) $$

for all $a,b\in I$ and $t\in [0,1]$. Function *f* is called concave if −*f* is convex.

The Hermite–Hadamard inequality [4] for convex functions $f:I\rightarrow \mathbb{R}$ on an interval of real line is defined as

2 $$ f\biggl(\frac{a+b}{2}\biggr)\leq \frac{1}{b-a} \int^{b}_{a}f(x)\,dx\leq \frac{f(a)+f(b)}{2}, $$

where $a,b\in I$ with $a< b$.

Since the Hermite–Hadamard inequality has many applications, many authors generalized this inequality. The Hermite–Hadamard inequality is also established for several kinds of convex functions. For more results and generalizations, see [2, 6, 10–14]. The Hermite–Hadamard inequality (2) is not only established for the classical integral but also for fractional integrals (e.g., see [1, 7, 18, 22]), for conformable fractional integrals (e.g., see [19, 21]), and recently for generalized fractional integrals (e.g., see [8, 9]).

### Definition 1.1

([5])

Let $s\in (0,1]$. A function $f:I\subset \mathbb{R}_{+}\rightarrow \mathbb{R}$, where $\mathbb{R}_{+}=[0,\infty)$, is called *s*-convex function in the second sense if

3 $$ f\bigl(ta+(1-t)b\bigr)\leq t^{s}f(a)+(1-t)^{s}f(b) $$

for all $a,b\in I$ and $t\in [0,1]$.

### Definition 1.2

([3, 23])

A function $f:[0,b]\rightarrow \mathbb{R}$, with $b>0$, is said to be *m*-convex if the following inequality holds:

4 $$ f\bigl(ta+m(1-t)c\bigr)\leq tf(a)+m(1-t)f(c) $$

for all $a,c\in [0,b]$ and $t\in [0,1]$ and for all $m\in [0,1]$. *f* is *m*-concave if −*f* is *m*-convex.

### Definition 1.3

([15])

Let $\alpha >0$ with $n-1<\alpha \leq n$, $n\in \mathbb{N}$, and $1< x< b$. The left- and right-hand side Riemann–Liouville fractional integrals of order *α* of function *f* are given by

$$ J^{\alpha }_{a+}f(x)=\frac{1}{\Gamma (\alpha)} \int_{a}^{x}(x-t)^{ \alpha -1}f(t)\,dt, $$

and

$$ J^{\alpha }_{b-}f(x)=\frac{1}{\Gamma (\alpha)} \int_{x}^{b}(t-x)^{ \alpha -1}f(t)\,dt, $$

respectively, where $\Gamma (\alpha)$ is the gamma function defined by $\Gamma (\alpha)=\int_{0}^{\infty }e^{-t}t^{\alpha -1}\,dt$.

### Definition 1.4

([16])

Let $\alpha >0$ with $n-1<\alpha \leq n$, $n\in \mathbb{N}$, and $1< x< b$. The left- and right-hand side Hadamard fractional integrals of order *α* of function *f* are given by

$$ H^{\alpha }_{a+}f(x)=\frac{1}{\Gamma (\alpha)} \int_{a}^{x} \biggl( \ln \frac{x}{t} \biggr) ^{\alpha -1}\frac{f(t)}{t}\,dt, $$

and

$$ H^{\alpha }_{b-}f(x)=\frac{1}{\Gamma (\alpha)} \int_{x}^{b} \biggl( \ln \frac{t}{x} \biggr) ^{\alpha -1}\frac{f(t)}{t}\,dt. $$

### Definition 1.5

([9])

Let $[a,b]\subset \mathbb{R}$ be a finite interval. Then the left- and right-hand side Katugampola fractional integrals of order $\alpha (>0)$ of $f\in X^{p}_{c}(a,b)$ are defined by

$$ {}^{\rho }I^{\alpha }_{a+}f(x)= \frac{\rho^{1-\alpha }}{\Gamma (\alpha)} \int_{a}^{x}\bigl(x^{\rho }-t^{ \rho } \bigr)^{\alpha -1}t^{\rho -1}f(t)\,dt $$

and

$$ {}^{\rho }I^{\alpha }_{b-}f(x)= \frac{\rho^{1-\alpha }}{\Gamma (\alpha)} \int_{x}^{b}\bigl(t^{\rho }-x^{ \rho } \bigr)^{\alpha -1}t^{\rho -1}f(t)\,dt, $$

with $a< x< b$ and $\rho >0$, where $X^{p}_{c}(a,b)$ ($c\in \mathbb{R}$, $1\leq p\leq \infty $ ) is the space of those complex-valued Lebesgue measurable functions *f* on $[a,b]$ for which $\|f\|_{X^{p}_{c}}<\infty $, where the norm is defined by

$$ \Vert f \Vert _{X^{p}_{c}}= \biggl( \int_{a}^{b} \bigl\vert t^{c}f(t) \bigr\vert ^{p}\frac{dt}{t} \biggr) ^{1/p}< \infty $$

for $1\leq p<\infty $, $c\in \mathbb{R}$ and for the case $p=\infty $,

$$ \Vert f \Vert _{X^{\infty }_{c}}= \mathop{\operatorname{ess\,sup}} _{a\leq t\leq b}\bigl[t^{c} \bigl\vert f(t) \bigr\vert \bigr], $$

where ess sup stands for essential supremum.

### Theorem 1.6

([9])

*Let* $\alpha >0$ *and* $\rho >0$. *Then*, *for* $x>a$,

1. $\lim_{\rho \rightarrow 1} ^{\rho }I^{\alpha }_{a+}f(x)=J^{ \alpha }_{a+}f(x)$,
2. $\lim_{\rho \rightarrow 0+} ^{\rho }I^{\alpha }_{a+}f(x)=H^{ \alpha }_{a+}f(x)$.

### Lemma 1.7

([20])

*For* $0<\alpha \leq 1$ *and* $0\leq a< b$, *we have*

$$ \bigl\vert a^{\alpha }-b^{\alpha } \bigr\vert \leq (b-a)^{\alpha }. $$

We recall the classical beta functions:

$$ \beta (a,b)= \int_{0}^{1}x^{a-1}(1-x)^{b-1} \,dx. $$

We introduce the following generalization of beta function:

$$ ^{\rho }\gamma (a,b)= \int_{0}^{1}\bigl(x^{\rho } \bigr)^{a-1}\bigl(1-x^{\rho }\bigr)^{b-1}x ^{\rho -1}\,dx. $$

Note that as $\rho \rightarrow 1$ then $^{\rho }\gamma (a,b)\rightarrow \beta (a,b)$.

In this paper, we give the Hermite–Hadamard type inequalities for *s*-convex functions and for *m*-convex functions via generalized fractional integral. Throughout the paper, $X^{p}_{c}(a,b)$ ($c \in \mathbb{R}$, $1\leq p\leq \infty $) is the space as defined in Definition 1.5 and $L_{1}[a,b]$ stands for the space of Lebesgue integrable over the closed interval $[a,b]$ where *a*, *b* are some real numbers with $a< b$.

## Hermite–Hadamard type inequalities for *s*-convex function

In this section we give Hermite–Hadamard type inequalities for *s*-convex function.

### Theorem 2.1

*Let* $\alpha >0$ *and* $\rho >0$. *Let* $f:[a^{\rho },b^{\rho }]\subset \mathbb{R}_{+}\rightarrow \mathbb{R}$ *be a positive function with* $0\leq a< b$ *and* $f\in X^{p}_{c}(a^{\rho },b^{\rho })$. *If* *f* *is also an* *s*-*convex function on* $[a^{\rho },b^{\rho }]$, *then the following inequalities hold*:

5 $$\begin{aligned} 2^{s-1}f \biggl( \frac{a^{\rho }+b^{\rho }}{2} \biggr) &\leq \frac{\rho^{\alpha }\Gamma (\alpha +1)}{2(b^{\rho }+a^{\rho })^{ \alpha }} \bigl[ {}^{\rho }I^{\alpha }_{a+}f \bigl(b^{\rho }\bigr)+{}^{\rho }I^{ \alpha }_{b-}f \bigl(a^{\rho }\bigr) \bigr] \\ & \leq \biggl[ \frac{\alpha }{\alpha +s}+\alpha \beta (\alpha,s+1) \biggr] \frac{f(a^{\rho })+f(b^{\rho })}{2}, \end{aligned}$$

*where the fractional integrals are considered for the function* $f(x^{\rho })$ *and evaluated at* *a* *and* *b*, *respectively*.

### Proof

Let $t\in [0,1]$. Consider $x,y\in [a,b]$, $a\geq 0$, defined by $x^{\rho }=t^{\rho }a^{\rho }+(1-t^{\rho })b^{\rho }$, $y^{\rho }=t ^{\rho }b^{\rho }+(1-t^{\rho })a^{\rho }$. Since *f* is an *s*-convex function on $[a^{\rho },b^{\rho }]$, we have

$$ f \biggl( \frac{x^{\rho }+y^{\rho }}{2} \biggr) \leq \frac{f(x^{\rho })+f(y ^{\rho })}{2^{s}}. $$

Then we have

6 $$ 2^{s}f \biggl( \frac{a^{\rho }+b^{\rho }}{2} \biggr) \leq f \bigl(t^{\rho }a^{ \rho }+\bigl(1-t^{\rho } \bigr)b^{\rho }\bigr)+f\bigl(t^{\rho }b^{\rho }+ \bigl(1-t^{\rho }\bigr)a ^{\rho }\bigr). $$

Multiplying both sides of (6) by $t^{\alpha \rho -1}$, $\alpha >0$ and then integrating the resulting inequality with respect to *t* over $[0,1]$, we obtain

7 $$\begin{aligned} \frac{2^{s}}{\alpha \rho }f \biggl( \frac{a^{\rho }+b^{\rho }}{2} \biggr) \leq& \int^{1}_{0}t^{\alpha \rho -1}f\bigl(t^{\rho }a^{\rho }+ \bigl(1-t^{\rho }\bigr)b ^{\rho }\bigr)\,dt + \int^{1}_{0}t^{\alpha \rho -1}f\bigl(t^{\rho }b^{\rho }+ \bigl(1-t ^{\rho }\bigr)a^{\rho }\bigr)\,dt \\ =& \int_{b}^{a} \biggl( \frac{b^{\rho }-x^{\rho }}{b^{\rho }-a^{\rho }} \biggr) ^{\alpha -1}f\bigl(x^{\rho }\bigr)\frac{x^{\rho -1}}{a^{\rho }-b^{\rho }}\,dx \\ &{}+ \int_{a}^{b} \biggl( \frac{y^{\rho }-a^{\rho }}{b^{\rho }-a^{\rho }} \biggr) ^{\alpha -1}f\bigl(y^{\rho }\bigr)\frac{y^{\rho -1}}{b^{\rho }-a^{\rho }}\,dy \\ =&\frac{\rho^{\alpha -1}\Gamma (\alpha)}{(b^{\rho }+a^{\rho })^{ \alpha }} \bigl[ {}^{\rho }I^{\alpha }_{a+}f \bigl(b^{\rho }\bigr)+{}^{\rho }I^{ \alpha }_{b-}f \bigl(a^{\rho }\bigr) \bigr] . \end{aligned}$$

This establishes the first inequality. For the proof of the second inequality in (5), we first observe that for an *s*-convex function *f*, we have

$$ f\bigl(t^{\rho }a^{\rho }+\bigl(1-t^{\rho } \bigr)b^{\rho }\bigr)\leq \bigl( t^{\rho } \bigr) ^{s}f \bigl(a^{\rho }\bigr)+ \bigl( 1-t^{\rho } \bigr) ^{s}f \bigl(b^{\rho }\bigr) $$

and

$$ f\bigl(t^{\rho }b^{\rho }+\bigl(1-t^{\rho } \bigr)a^{\rho }\bigr)\leq \bigl( t^{\rho } \bigr) ^{s}f \bigl(b^{\rho }\bigr)+ \bigl( 1-t^{\rho } \bigr) ^{s}f \bigl(a^{\rho }\bigr). $$

By adding these inequalities, we get

8 $$ f\bigl(t^{\rho }a^{\rho }+\bigl(1-t^{\rho } \bigr)b^{\rho }\bigr)+f\bigl(t^{\rho }b^{\rho }+\bigl(1-t ^{\rho }\bigr)a^{\rho }\bigr)\leq \bigl( \bigl( t^{\rho } \bigr) ^{s}+ \bigl( 1-t ^{\rho } \bigr) ^{s} \bigr) \bigl[ f\bigl(a^{\rho }\bigr)+f\bigl(b^{\rho }\bigr) \bigr] . $$

Multiplying both sides of (8) by $t^{\alpha \rho -1}$, $\alpha >0$ and then integrating the resulting inequality with respect to *t* over $[0,1]$, we obtain

9 $$\begin{aligned}& \frac{\rho^{\alpha -1}\Gamma (\alpha)}{(b^{\rho }+a^{\rho })^{\alpha }} \bigl[ {}^{\rho }I^{\alpha }_{a+}f \bigl(b^{\rho }\bigr)+{}^{\rho }I^{\alpha }_{b-}f\bigl(a ^{\rho }\bigr) \bigr] \\& \quad \leq \int^{1}_{0}t^{\alpha \rho -1} \bigl( \bigl( t^{ \rho } \bigr) ^{s}+ \bigl( 1-t^{\rho } \bigr) ^{s} \bigr) \bigl[ f\bigl(a^{ \rho }\bigr)+f\bigl(b^{\rho } \bigr) \bigr]\,dt. \end{aligned}$$

Since

$$ \int^{1}_{0}t^{\alpha \rho +s\rho -1}\,dt= \frac{1}{\rho (\alpha +s)}, $$

and by choosing the change of variable $t^{\rho }=z$, we have

$$ \int^{1}_{0}t^{\alpha \rho -1} \bigl( 1-t^{\rho } \bigr) ^{s}\,dt= \frac{ \beta (\alpha,s+1)}{\rho }. $$

Thus (9) becomes

10 $$ \frac{\rho^{\alpha -1}\Gamma (\alpha)}{(b^{\rho }+a^{\rho })^{\alpha }} \bigl[ {}^{\rho }I^{\alpha }_{a+}f \bigl(b^{\rho }\bigr)+{}^{\rho }I^{\alpha }_{b-}f\bigl(a ^{\rho }\bigr) \bigr] \leq \frac{1}{\rho } \biggl[ \frac{1}{\alpha +s}+ \beta ( \alpha,s+1) \biggr] \bigl( f\bigl(a^{\rho }\bigr)+f \bigl(b^{\rho }\bigr) \bigr). $$

Thus (7) and (10) give (5). □

### Remark 2.2

By letting $\rho \rightarrow 1$ in (5) of Theorem 2.1, we get Theorem 3 of [22].

### Theorem 2.3

*Let* $\alpha >0$ *and* $\rho >0$. *Let* $f:[a^{\rho },b^{\rho }]\subset \mathbb{R}_{+}\rightarrow \mathbb{R}$ *be a differentiable mapping on* $(a^{\rho },b^{\rho })$ *with* $0\leq a< b$. *If* $|f'|$ *is* *s*-*convex on* $[a^{\rho },b^{\rho }]$, *then the following inequality holds*:

11 $$\begin{aligned}& \biggl\vert \frac{f(a^{\rho })+f(b^{\rho })}{2}-\frac{\rho^{\alpha }\Gamma (\alpha +1)}{2(b^{\rho }+a^{\rho })^{\alpha }} \bigl[{} ^{\rho }I^{ \alpha }_{a+}f\bigl(b^{\rho } \bigr)+{}^{\rho }I^{\alpha }_{b-}f\bigl(a^{\rho }\bigr) \bigr] \biggr\vert \\& \quad \leq \frac{b^{\rho }-a^{\rho }}{2} \biggl[ \frac{1}{\alpha +s+1}+ \beta (\alpha +1,s+1) \biggr] \bigl( \bigl\vert f'\bigl(a^{\rho }\bigr) \bigr\vert + \bigl\vert f'\bigl(b^{\rho }\bigr) \bigr\vert \bigr). \end{aligned}$$

### Proof

From (7) one can have

12 $$\begin{aligned} &\frac{\rho^{\alpha -1}\Gamma (\alpha)}{(b^{\rho }+a^{\rho })^{ \alpha }} \bigl[ {}^{\rho }I^{\alpha }_{a+}f \bigl(b^{\rho }\bigr)+{}^{\rho }I^{ \alpha }_{b-}f \bigl(a^{\rho }\bigr) \bigr] \\ &\quad = \int^{1}_{0}t^{\alpha \rho -1}f\bigl(t^{\rho }a^{\rho }+ \bigl(1-t^{\rho }\bigr)b ^{\rho }\bigr)\,dt + \int^{1}_{0}t^{\alpha \rho -1}f\bigl(t^{\rho }b^{\rho }+ \bigl(1-t ^{\rho }\bigr)a^{\rho }\bigr)\,dt. \end{aligned}$$

Integrating by parts, we get

13 $$\begin{aligned}& \frac{f(a^{\rho })+f(b^{\rho })}{\alpha \rho }-\frac{\rho^{\alpha -1} \Gamma (\alpha)}{(b^{\rho }+a^{\rho })^{\alpha }} \bigl[ {}^{\rho }I ^{\alpha }_{a+}f\bigl(b^{\rho }\bigr)+{}^{\rho }I^{\alpha }_{b-}f \bigl(a^{\rho }\bigr) \bigr] \\& \quad =\frac{b^{\rho }-a^{\rho }}{\alpha } \int_{0}^{1}t^{\rho (\alpha +1)-1} \bigl[ f'\bigl(t^{\rho }b^{\rho }+\bigl(1-t^{\rho } \bigr)a^{\rho }\bigr)-f'\bigl(t^{\rho }a^{ \rho }+ \bigl(1-t^{\rho }\bigr)b^{\rho }\bigr) \bigr]\,dt. \end{aligned}$$

By using the triangle inequality and *s*-convexity of $|f'|$ and the change of variable $t^{\rho }=z$, we obtain

14 $$\begin{aligned} & \biggl\vert \frac{f(a^{\rho })+f(b^{\rho }}{\alpha \rho }-\frac{ \rho^{\alpha -1}\Gamma (\alpha)}{(b^{\rho }+a^{\rho })^{\alpha }} \bigl[{} ^{\rho }I^{\alpha }_{a+}f\bigl(b^{\rho } \bigr)+{}^{\rho }I^{\alpha }_{b-}f\bigl(a ^{\rho }\bigr) \bigr] \biggr\vert \\ &\quad \leq \frac{b^{\rho }-a^{\rho }}{\alpha } \int_{0}^{1}t^{\rho (\alpha +1)-1} \bigl\vert \bigl[ f'\bigl(t^{\rho }b^{\rho }+\bigl(1-t^{\rho } \bigr)a^{\rho }\bigr) -f'\bigl(t ^{\rho }a^{\rho }+ \bigl(1-t^{\rho }\bigr)b^{\rho }\bigr) \bigr] \bigr\vert \,dt \\ &\quad \leq \frac{b^{\rho }-a^{\rho }}{\alpha } \int_{0}^{1}t^{\rho (\alpha +1)-1} \bigl[ \bigl\vert f'\bigl(t^{\rho }b^{\rho }+\bigl(1-t^{\rho } \bigr)a^{\rho }\bigr) \bigr\vert + \bigl\vert f'\bigl(t ^{\rho }a^{\rho }+\bigl(1-t^{\rho }\bigr)b^{\rho } \bigr) \bigr\vert \bigr]\,dt \\ &\quad \leq \frac{b^{\rho }-a^{\rho }}{\alpha } \int_{0}^{1}t^{\rho (\alpha +1)-1} \bigl[ \bigl(t^{\rho }\bigr)^{s} \bigl\vert f' \bigl(b^{\rho }\bigr) \bigr\vert +\bigl(1-t^{\rho } \bigr)^{s} \bigl\vert f'\bigl(a^{\rho }\bigr) \bigr\vert \\ &\qquad {}+\bigl(t^{\rho }\bigr)^{s} \bigl\vert f'\bigl(a^{\rho }\bigr) \bigr\vert +\bigl(1-t^{\rho } \bigr)^{s} \bigl\vert f'\bigl(b^{\rho }\bigr) \bigr\vert \bigr]\,dt \\ &\quad =\frac{b^{\rho }-a^{\rho }}{\alpha } \int_{0}^{1}t^{\rho (\alpha +1)-1} \bigl[ \bigl(t^{\rho }\bigr)^{s}+\bigl(1-t^{\rho } \bigr)^{s} \bigr] \bigl[ \bigl\vert f' \bigl(a^{\rho }\bigr) \bigr\vert + \bigl\vert f'\bigl(b ^{\rho }\bigr) \bigr\vert \bigr]\,dt \\ &\quad =\frac{b^{\rho }-a^{\rho }}{\alpha \rho } \biggl[ \frac{1}{\alpha +s+1}+ \beta (\alpha +1,s+1) \biggr] \bigl[ \bigl\vert f'\bigl(a^{\rho }\bigr) \bigr\vert + \bigl\vert f'\bigl(b^{\rho }\bigr) \bigr\vert \bigr] . \end{aligned}$$

 □

### Corollary 2.4

*Under the same assumptions of Theorem *2.3.

1. *If* $\rho =1$, *then*
  15 $$\begin{aligned} & \biggl\vert \frac{f(a)+f(b)}{2}- \frac{\Gamma (\alpha +1)}{2(b+a)^{\alpha }} \bigl[ J^{\alpha }_{a+}f(b)+J ^{\alpha }_{b-}f(a) \bigr] \biggr\vert \\ &\quad \leq \frac{b-a}{2} \biggl[ \frac{1}{\alpha +s+1}+\beta (\alpha +1,s+1) \biggr] \bigl( \bigl\vert f'(a) \bigr\vert + \bigl\vert f'(b) \bigr\vert \bigr). \end{aligned}$$
2. *If* $\rho =s=1$, *then*
  16 $$\begin{aligned} & \biggl\vert \frac{f(a)+f(b)}{2}- \frac{\Gamma (\alpha +1)}{2(b+a)^{\alpha }} \bigl[ J^{\alpha }_{a+}f(b)+J ^{\alpha }_{b-}f(a) \bigr] \biggr\vert \\ &\quad \leq \frac{b-a}{2} \biggl[ \frac{1}{\alpha +2}+\beta (\alpha +1,2) \biggr] \bigl( \bigl\vert f'(a) \bigr\vert + \bigl\vert f'(b) \bigr\vert \bigr). \end{aligned}$$
3. *If* $\rho =s=\alpha =1$, *then*
  17 $$ \biggl\vert \frac{f(a)+f(b)}{2}-\frac{1}{b+a} \int_{a}^{b}f(x)\,dx \biggr\vert \leq \frac{b-a}{4} \bigl( \bigl\vert f'(a) \bigr\vert + \bigl\vert f'(b) \bigr\vert \bigr). $$

In order to prove our further results, we need the following lemma.

### Lemma 2.5

*Let* $\alpha >0$ *and* $\rho >0$. *Let* $f:[a^{\rho },b^{\rho }]\subset \mathbb{R}_{+}\rightarrow \mathbb{R}$ *be a differentiable mapping on* $(a^{\rho },b^{\rho })$ *with* $0\leq a< b$. *Then the following equality holds if the fractional integrals exist*:

18 $$\begin{aligned}& \frac{f(a^{\rho })+f(b^{\rho })}{2}-\frac{\rho^{\alpha }\Gamma ( \alpha +1)}{2(b^{\rho }+a^{\rho })^{\alpha }} \bigl[ {}^{\rho }I^{\alpha }_{a+}f \bigl(b^{\rho }\bigr)+{}^{\rho }I^{\alpha }_{b-}f \bigl(a^{\rho }\bigr) \bigr] \\& \quad =\frac{\rho (b^{\rho }-a^{\rho })}{2} \int_{0}^{1} \bigl[ \bigl(1-t^{\rho } \bigr)^{ \alpha }-\bigl(t^{\rho }\bigr)^{\alpha } \bigr] t^{\rho -1}f'\bigl(t^{\rho }a^{ \rho }+ \bigl(1-t^{\rho }\bigr)b^{\rho }\bigr)\,dt. \end{aligned}$$

### Proof

By using the similar arguments as in the proof of Lemma 2 in [18]. First consider

19 $$\begin{aligned} & \int_{0}^{1}\bigl(1-t^{\rho } \bigr)^{\alpha }t^{\rho -1}f'\bigl(t^{\rho }a^{\rho }+ \bigl(1-t ^{\rho }\bigr)b^{\rho }\bigr)\,dt \\ &\quad =\frac{(1-t^{\rho })^{\alpha }f(t^{\rho }a^{\rho }+(1-t^{\rho })b ^{\rho })}{\rho (a^{\rho }-b^{\rho })}\bigg|_{0}^{1} \\ &\qquad {} +\frac{\alpha }{a ^{\rho }-b^{\rho }} \int_{0}^{1}\bigl(1-t^{\rho } \bigr)^{\alpha -1}t^{\rho -1}f\bigl(t ^{\rho }a^{\rho }+ \bigl(1-t^{\rho }\bigr)b^{\rho }\bigr)\,dt \\ &\quad =\frac{f(b^{\rho })}{\rho (b^{\rho }-a^{\rho })}-\frac{\alpha }{b ^{\rho }-a^{\rho }} \int_{b}^{a} \biggl( \frac{x^{\rho }-a^{\rho }}{b ^{\rho }-a^{\rho }} \biggr) ^{\alpha -1}\cdot \frac{x^{\rho -1}}{a^{ \rho }-b^{\rho }}\,dx \\ &\quad =\frac{f(b^{\rho })}{\rho (b^{\rho }-a^{\rho })}-\frac{\rho^{\alpha -1}\Gamma (\alpha +1)}{(b^{\rho }-a^{\rho })^{\alpha +1}}\cdot{}^{ \rho }I_{b-}^{\alpha }f \bigl(x^{\rho }\bigr)\bigg|_{x=a}. \end{aligned}$$

Similarly, we can show that

20 $$\begin{aligned}& \int_{0}^{1}t^{\rho \alpha }\cdot t^{\rho -1}f'\bigl(t^{\rho }a^{\rho }+\bigl(1-t ^{\rho }\bigr)b^{\rho }\bigr)\,dt \\& \quad = - \frac{f(a^{\rho })}{\rho (b^{\rho }-a^{\rho })}+ \frac{\rho^{\alpha -1} \Gamma (\alpha +1)}{(b^{\rho }-a^{\rho })^{\alpha +1}}\cdot^{\rho }I _{a+}^{\alpha }f \bigl(x^{\rho }\bigr)\bigg|_{x=b}. \end{aligned}$$

Thus from (19) and (20) we get (18). □

### Remark 2.6

By taking $\rho =1$ in (18) of Lemma 2.5, we get Lemma 2 in [17].

Throughout all other results we denote

$$ I_{f}(\alpha,\rho,a,b)=\frac{f(a^{\rho })+f(b^{\rho })}{2}-\frac{ \rho^{\alpha }\Gamma (\alpha +1)}{2(b^{\rho }+a^{\rho })^{\alpha }} \bigl[ {}^{\rho }I^{\alpha }_{a+}f\bigl(b^{\rho } \bigr)+{}^{\rho }I^{\alpha }_{b-}f\bigl(a ^{\rho }\bigr) \bigr] . $$

### Theorem 2.7

*Let* $\alpha >0$ *and* $\rho >0$. *Let* $f:[a^{\rho },b^{\rho }]\subset \mathbb{R}_{+}\rightarrow \mathbb{R}$ *be a differentiable mapping on* $(a^{\rho },b^{\rho })$ *such that* $f'\in L_{1}[a,b]$ *with* $0\leq a< b$. *If* $|f'|^{q}$ *is* *s*-*convex on* $[a^{\rho },b^{\rho }]$ *for some fixed* $q\geq 1$, *then the following inequality holds*:

21 $$\begin{aligned} \bigl\vert I_{f}(\alpha,\rho,a,b) \bigr\vert \leq{}& \frac{\rho (b^{\rho }-a ^{\rho })}{2}\biggl(\frac{1}{\rho (\alpha +1)}\biggr)^{1-1/q} \\ &{}\times \biggl( \biggl( {}^{\rho }\gamma (s+1,\alpha +1)+ \frac{1}{\rho ( \alpha +s+1)} \biggr) \bigl\vert f'\bigl(a^{\rho } \bigr) \bigr\vert ^{q} \\ &{}+ \bigl( {}^{\rho }\gamma (1,\alpha +s+1)+ {}^{\rho }\gamma (\alpha +1,s+1) \bigr) \bigl\vert f'\bigl(b^{\rho }\bigr) \bigr\vert ^{q} \biggr)^{1/q}. \end{aligned}$$

### Proof

Using Lemma 2.5 and the power mean inequality and *s*-convexity of $|f'|^{q}$, we obtain

22 $$\begin{aligned} & \bigl\vert I_{f}(\alpha,\rho,a,b) \bigr\vert \\ &\quad = \biggl\vert \frac{\rho (b^{\rho }-a^{\rho })}{2} \int_{0}^{1} \bigl\{ \bigl(1-t ^{\rho } \bigr)^{\alpha }-\bigl(t^{\rho }\bigr)^{\alpha } \bigr\} t^{\rho -1}f'\bigl(t^{ \rho }a^{\rho }+ \bigl(1-t^{\rho }\bigr)b^{\rho }\bigr)\,dt \biggr\vert \\ &\quad \leq \frac{\rho (b^{\rho }-a^{\rho })}{2} \biggl( \int_{0}^{1} \bigl\vert \bigl(1-t ^{\rho } \bigr)^{\alpha }-\bigl(t^{\rho }\bigr)^{\alpha } \bigr\vert t^{\rho -1}\,dt \biggr) ^{1-1/q} \\ &\qquad {}\times \biggl( \int_{0}^{1} \bigl\vert \bigl(1-t^{\rho } \bigr)^{\alpha }-\bigl(t^{\rho }\bigr)^{ \alpha } \bigr\vert t^{\rho -1} \bigl\vert f'\bigl(t^{\rho }a^{\rho }+ \bigl(1-t^{\rho }\bigr)b^{ \rho }\bigr) \bigr\vert ^{q} \,dt \biggr) ^{1/q} \\ &\quad \leq \frac{\rho (b^{\rho }-a^{\rho })}{2} \biggl( \int_{0}^{1} \bigl\{ \bigl(1-t ^{\rho } \bigr)^{\alpha }+\bigl(t^{\rho }\bigr)^{\alpha } \bigr\} t^{\rho -1}\,dt \biggr) ^{1-1/q} \\ &\qquad {}\times \biggl( \int_{0}^{1} \bigl\{ \bigl(1-t^{\rho } \bigr)^{\alpha }+\bigl(t^{\rho }\bigr)^{ \alpha } \bigr\} t^{\rho -1} \bigl[\bigl(t^{\rho }\bigr)^{s} \bigl\vert f'\bigl(a^{\rho }\bigr) \bigr\vert ^{q}+ \bigl(1-t ^{\rho }\bigr)^{s} \bigl\vert f' \bigl(b^{\rho }\bigr) \bigr\vert ^{q} \bigr]\,dt \biggr) ^{1/q} \\ &\quad =\frac{\rho (b^{\rho }-a^{\rho })}{2}\biggl(\frac{1}{\rho (\alpha +1)} \biggr)^{1-1/q} \\ &\qquad {}\times \biggl( \biggl( {}^{\rho }\gamma (s+1,\alpha +1)+\frac{1}{\rho ( \alpha +s+1)} \biggr) \bigl\vert f'\bigl(a^{\rho }\bigr) \bigr\vert ^{q} \\ &\qquad {}+ \bigl( {}^{\rho }\gamma (1,\alpha +s+1)+ {}^{\rho }\gamma (\alpha +1,s+1) \bigr) \bigl\vert f'\bigl(b^{\rho }\bigr) \bigr\vert ^{q} \biggr)^{1/q}. \end{aligned}$$

Hence the proof is completed. □

### Corollary 2.8

*Under the similar conditions of Theorem *2.7.

1. *If* $\rho =1$, *then*
  $$\begin{aligned} & \biggl\vert \frac{f(a)+f(b)}{2}- \frac{\Gamma (\alpha +1)}{2(b+a)^{\alpha }} \bigl[ J^{\alpha }_{a+}f(b)+J ^{\alpha }_{b-}f(a) \bigr] \biggr\vert \\ &\quad \leq \frac{ (b-a)}{2}\biggl(\frac{1}{(\alpha +1)}\biggr)^{1-1/q} \times \biggl( \biggl( \beta (s+1,\alpha +1)+\frac{1}{(\alpha +s+1)} \biggr) \bigl\vert f'(a) \bigr\vert ^{q} \\ &\qquad {}+ \bigl(\beta (1,\alpha +s+1)+ \beta (\alpha +1,s+1) \bigr) \bigl\vert f'(b) \bigr\vert ^{q} \biggr)^{1/q}. \end{aligned}$$
2. *If* $\rho =s=1$, *then*
  $$\begin{aligned} & \biggl\vert \frac{f(a)+f(b)}{2}- \frac{\Gamma (\alpha +1)}{2(b+a)^{\alpha }} \bigl[ J^{\alpha }_{a+}f(b)+J ^{\alpha }_{b-}f(a) \bigr] \biggr\vert \\ &\quad \leq \frac{ (b-a)}{2}\biggl(\frac{1}{(\alpha +1)}\biggr)^{1-1/q} \times \biggl( \biggl( \beta (2,\alpha +1)+\frac{1}{(\alpha +2)} \biggr) \bigl\vert f'(a) \bigr\vert ^{q} \\ &\qquad {}+ \bigl(\beta (1,\alpha +2)+ \beta (\alpha +1,2) \bigr) \bigl\vert f'(b) \bigr\vert ^{q} \biggr)^{1/q}. \end{aligned}$$
3. *If* $\rho =s=\alpha =1$, *then*
  $$ \biggl\vert \frac{f(a)+f(b)}{2}-\frac{1}{b+a} \int_{a}^{b} f(x)\,dx \biggr\vert \leq \frac{ (b-a)}{2^{2-1/q}} \times \biggl( \frac{ \vert f'(a) \vert ^{q} + \vert f'(b) \vert ^{q}}{2} \biggr) ^{1/q}. $$

### Theorem 2.9

*Let* $\alpha >0$ *and* $\rho >0$. *Let* $f:[a^{\rho },b^{\rho }]\subset \mathbb{R}_{+}\rightarrow \mathbb{R}$ *be a differentiable mapping on* $(a^{\rho },b^{\rho })$ *such that* $f'\in L_{1}[a,b]$ *with* $0\leq a< b$. *If* $|f'|^{q}$ *is* *s*-*convex on* $[a^{\rho },b^{\rho }]$ *for some fixed* $q\geq 1$, *then the following inequality holds*:

23 $$\begin{aligned} & \bigl\vert I_{f}(\alpha, \rho,a,b) \bigr\vert \\ &\quad \leq \frac{\rho^{\frac{1}{q}}(b^{\rho }-a^{\rho })}{2} \biggl( \biggl[ \beta (s+1, \alpha +1)+ \frac{1}{\alpha +s+1} \biggr] \bigl[ \bigl\vert f' \bigl(a^{\rho }\bigr) \bigr\vert ^{q}+ \bigl\vert f'\bigl(b^{ \rho }\bigr) \bigr\vert ^{q}\bigr] \biggr) ^{1/q}. \end{aligned}$$

### Proof

Using Lemma 2.5, the property of modulus, the power mean inequality, and the fact that $|f'|^{q}$ is an *s*-convex function, we have

24 $$\begin{aligned} & \bigl\vert I_{f}(\alpha, \rho,a,b) \bigr\vert \\ &\quad \leq \biggl\vert \frac{\rho (b^{\rho }-a^{\rho })}{2} \int_{0}^{1} \bigl\{ \bigl(1-t ^{\rho } \bigr)^{\alpha }-\bigl(t^{\rho }\bigr)^{\alpha } \bigr\} t^{\rho -1} \bigl\vert f'\bigl(t ^{\rho }a^{\rho }+ \bigl(1-t^{\rho }\bigr)b^{\rho }\bigr) \bigr\vert \,dt \biggr\vert \\ &\quad \leq \frac{\rho (b^{\rho }-a^{\rho })}{2} \biggl( \int_{0}^{1}t^{ \rho -1}\,dt \biggr) ^{1-1/q} \\ &\qquad {}\times \biggl( \int_{0}^{1} \bigl\{ \bigl(1-t^{\rho } \bigr)^{ \alpha }-\bigl(t^{\rho }\bigr)^{\alpha } \bigr\} \bigl\vert f'\bigl(t^{\rho }a^{\rho }+\bigl(1-t ^{\rho }\bigr)b^{\rho }\bigr) \bigr\vert ^{q}\,dt \biggr) ^{1/q} \\ &\quad \leq \frac{\rho (b^{\rho }-a^{\rho })}{2}\frac{1}{\rho^{1-1/q}} \\ &\qquad {}\times \biggl( \int_{0}^{1} \bigl\{ \bigl(1-t^{\rho } \bigr)^{\alpha }+\bigl(t^{\rho }\bigr)^{\alpha } \bigr\} \bigl[ \bigl(t^{\rho }\bigr)^{s} \bigl\vert f' \bigl(a^{\rho }\bigr) \bigr\vert ^{q}+\bigl(1-t^{\rho } \bigr)^{s} \bigl\vert f'\bigl(b ^{\rho }\bigr) \bigr\vert ^{q} \bigr]\,dt \biggr) ^{1/q} \\ &\quad =\frac{\rho^{\frac{1}{q}}(b^{\rho }-a^{\rho })}{2} \biggl( \bigl\vert f' \bigl(a^{ \rho }\bigr) \bigr\vert ^{q} \int_{0}^{1} \bigl\{ \bigl(1-t^{\rho } \bigr)^{\alpha }\bigl(t^{\rho }\bigr)^{s} + \bigl(t^{\rho }\bigr)^{\alpha }\bigl(t^{\rho } \bigr)^{s} \bigr\} \,dt \\ &\qquad {}+ \bigl\vert f'\bigl(b^{\rho }\bigr) \bigr\vert ^{q} \int_{0}^{1} \bigl\{ \bigl(1-t^{\rho } \bigr)^{\alpha }\bigl(1-t ^{\rho }\bigr)^{s} + \bigl(t^{\rho }\bigr)^{\alpha }\bigl(1-t^{\rho } \bigr)^{s} \bigr\} \,dt \biggr)^{1/q} \\ &\quad =\frac{\rho^{\frac{1}{q}}(b^{\rho }-a^{\rho })}{2} \bigl( A \bigl\vert f' \bigl(a^{ \rho }\bigr) \bigr\vert ^{q}+B \bigl\vert f'\bigl(b^{\rho }\bigr) \bigr\vert ^{q} \bigr) ^{1/q}. \end{aligned}$$

By using the change of variable $t^{\rho }=z$, we get

$$ A= \int_{0}^{1} \bigl\{ \bigl(1-t^{\rho } \bigr)^{\alpha }\bigl(t^{\rho }\bigr)^{s} + \bigl(t^{ \rho }\bigr)^{\alpha }\bigl(t^{\rho } \bigr)^{s} \bigr\} \,dt=\beta (s+1,\alpha +1)+\frac{1}{ \alpha +s+1} $$

and

$$ B= \int_{0}^{1} \bigl\{ \bigl(1-t^{\rho } \bigr)^{\alpha }\bigl(1-t^{\rho }\bigr)^{s} + \bigl(t^{ \rho }\bigr)^{\alpha }\bigl(1-t^{\rho } \bigr)^{s} \bigr\} \,dt=\beta (\alpha +1,s+1)+\frac{1}{ \alpha +s+1}. $$

Thus substituting the values of *A* and *B* in (24) and applying the fact that $\beta (a,b)=\beta (b,a)$, we get the desired result. □

### Corollary 2.10

*Under the similar conditions of Theorem *2.7.

1. *If* $\rho =1$, *then*
  $$\begin{aligned} & \biggl\vert \frac{f(a)+f(b)}{2}- \frac{\Gamma (\alpha +1)}{2(b+a)^{\alpha }} \bigl[ J^{\alpha }_{a+}f(b)+J ^{\alpha }_{b-}f(a) \bigr] \biggr\vert \\ &\quad \leq \frac{(b-a)}{2} \biggl( \biggl[ \beta (s+1,\alpha +1)+ \frac{1}{ \alpha +s+1} \biggr] \bigl[ \bigl\vert f'(a) \bigr\vert ^{q}+ \bigl\vert f'(b) \bigr\vert ^{q} \bigr] \biggr) ^{1/q}. \end{aligned}$$
2. *If* $\rho =s=1$, *then*
  $$\begin{aligned} & \biggl\vert \frac{f(a)+f(b)}{2}- \frac{\Gamma (\alpha +1)}{2(b+a)^{\alpha }} \bigl[ J^{\alpha }_{a+}f(b)+J ^{\alpha }_{b-}f(a) \bigr] \biggr\vert \\ &\quad \leq \frac{(b-a)}{2} \biggl( \biggl[ \beta (2,\alpha +1)+ \frac{1}{ \alpha +2} \biggr] \bigl[ \bigl\vert f'(a) \bigr\vert ^{q}+ \bigl\vert f'(b) \bigr\vert ^{q} \bigr] \biggr) ^{1/q}. \end{aligned}$$
3. *If* $\rho =s=\alpha =1$, *then*
  $$ \biggl\vert \frac{f(a)+f(b)}{2}-\frac{1}{b+a} \int_{a}^{b} f(x)\,dx \biggr\vert \leq \frac{(b-a)}{2} \biggl( \frac{ \vert f'(a) \vert ^{q}+ \vert f'(b) \vert ^{q}}{2} \biggr) ^{1/q}. $$

## Hermite–Hadamard type inequalities for *m*-convex function

In this section we give Hermite–Hadamard type inequalities for *m*-convex function.

### Theorem 3.1

*Let* $\alpha >0$ *and* $\rho >0$. *Let* $f:[a^{\rho },b^{\rho }]\subset \mathbb{R}_{+}\rightarrow \mathbb{R}$ *be a positive function with* $0\leq a< b$ *and* $f\in X^{p}_{c}(a^{\rho },b^{\rho })$. *If* *f* *is also an* *m*-*convex function on* $[a^{\rho },b^{\rho }]$, *then the following inequalities hold*:

25 $$\begin{aligned} f \biggl( \frac{m^{\rho }(a^{\rho }+b^{\rho })}{2} \biggr) & \leq \frac{\rho^{\alpha }\Gamma (\alpha +1)}{2((mb)^{\rho }-(ma)^{ \rho })^{\alpha }} {}^{\rho }I_{ma+}^{\alpha }f \bigl( (mb)^{\rho } \bigr) +\frac{m ^{\rho }\rho^{\alpha }\Gamma (\alpha +1)}{2(b^{\rho }-a^{\rho })^{ \alpha }} {}^{\rho }I_{b-}^{\alpha }f \bigl( a^{\rho } \bigr) \\ & \leq \frac{m^{\rho }}{2} \bigl( f\bigl(a^{\rho }\bigr)+f \bigl(b^{\rho }\bigr) \bigr). \end{aligned}$$

### Proof

Since *f* is *m*-convex, we have

$$ f \biggl( \frac{x^{\rho }+m^{\rho }y^{\rho }}{2} \biggr) \leq \frac{f(x ^{\rho })+m^{\rho }f(y^{\rho })}{2}. $$

Let $x^{\rho }=m^{\rho }t^{\rho }a^{\rho }+m^{\rho }(1-t^{\rho })b ^{\rho }$, $y^{\rho }=t^{\rho }b^{\rho }+(1-t^{\rho })a^{\rho }$ with $t\in [0,1]$. Then we obtain

26 $$ f \biggl( \frac{m^{\rho }(a^{\rho }+b^{\rho })}{2} \biggr) \leq \frac{f(m ^{\rho }t^{\rho }a^{\rho }+m^{\rho }(1-t^{\rho })b^{\rho }) +m^{ \rho }f(t^{\rho }b^{\rho }+(1-t^{\rho })a^{\rho })}{2}. $$

Multiplying both sides of (26) by $t^{\alpha \rho -1}$, $\alpha >0$ and then integrating the resulting inequality with respect to *t* over $[0,1]$, we obtain

27 $$\begin{aligned} &\frac{2}{\rho \alpha }f \biggl( \frac{m^{\rho }(a^{\rho }+b^{\rho })}{2} \biggr) \\ &\quad \leq \int_{0}^{1}t^{\alpha \rho -1}f\bigl(m^{\rho }t^{\rho }a^{\rho }+m ^{\rho }\bigl(1-t^{\rho }\bigr)b^{\rho }\bigr)\,dt+ m^{\rho } \int_{0}^{1}t^{\alpha \rho -1}f\bigl(t^{\rho }b^{\rho }+ \bigl(1-t^{\rho }\bigr)a^{\rho }\bigr)\,dt \\ &\quad = \int_{mb}^{ma} \biggl( \frac{x^{\rho }-(mb)^{\rho }}{(ma)^{\rho }-(mb)^{ \rho }} \biggr) ^{\alpha -1} x^{\rho -1}\frac{dx}{(ma)^{\rho }-(mb)^{ \rho }} \\ &\qquad {}+m^{\rho } \int_{a}^{b} \biggl( \frac{y^{\rho }-a^{\rho }}{b^{\rho }-a ^{\rho }} \biggr) ^{\alpha -1} y^{\rho -1} \frac{dy}{b^{\rho }-a^{\rho }} \\ &\quad =\frac{\rho^{\alpha -1}\Gamma (\alpha)}{((mb)^{\rho }-(ma)^{\rho })^{ \alpha }} {}^{\rho }I_{ma+}^{\alpha }f \bigl( (mb)^{\rho } \bigr) +\frac{m ^{\rho }\rho^{\alpha -1}\Gamma (\alpha)}{(b^{\rho }-a^{\rho })^{ \alpha }} {}^{\rho }I_{b-}^{\alpha }f \bigl( a^{\rho } \bigr). \end{aligned}$$

Now by multiplying both sides of (27) by $\frac{\alpha \rho }{2}$, we get the first inequality of (25). For the second inequality, using *m*-convexity of *f*, we have

28 $$ f\bigl(m^{\rho }t^{\rho }a^{\rho }+m^{\rho } \bigl(1-t^{\rho }\bigr)b^{\rho }\bigr) +m^{ \rho }f\bigl( \bigl(1-t^{\rho }\bigr)a^{\rho }+t^{\rho }b^{\rho } \bigr) \leq m^{\rho } \bigl[ f\bigl(a^{\rho }\bigr)+f \bigl(b^{\rho }\bigr) \bigr] . $$

Multiplying both sides of (28) by $t^{\alpha \rho -1}$, $\alpha >0$ and then integrating the resulting inequality with respect to *t* over $[0,1]$, we obtain

29 $$\begin{aligned} &\frac{\rho^{\alpha -1}\Gamma (\alpha)}{((mb)^{\rho }-(ma)^{\rho })^{ \alpha }} {}^{\rho }I_{ma+}^{\alpha }f \bigl( (mb)^{\rho } \bigr) +\frac{m ^{\rho }\rho^{\alpha -1}\Gamma (\alpha)}{(b^{\rho }-a^{\rho })^{ \alpha }} {}^{\rho }I_{b-}^{\alpha }f \bigl( a^{\rho } \bigr) \\ &\quad \leq \frac{m^{\rho }}{\rho \alpha } \bigl( f\bigl(a^{\rho }\bigr)+f \bigl(b^{\rho }\bigr) \bigr). \end{aligned}$$

Now, by multiplying both sides of (29) by $\frac{\alpha \rho }{2}$, we get the second inequality of (25). □

### Corollary 3.2

*Under the assumptions of Theorem *3.1, *we have*

1. *For* $\rho =1$, *then*
  30 $$\begin{aligned} &f \biggl( \frac{m(a+b)}{2} \biggr) \\ &\quad \leq \frac{\Gamma (\alpha +1)}{2(mb-ma)^{\alpha }} J_{ma+}^{\alpha }f(mb)+ \frac{m\Gamma (\alpha +1)}{2(b-a)^{\alpha }} J_{b-}^{\alpha }f ( a ) \\ &\quad \leq \frac{m}{2} \bigl( f(a)+f(b) \bigr). \end{aligned}$$
2. *For* $\rho =\alpha =1$, *then*
  31 $$\begin{aligned} f \biggl( \frac{m(a+b)}{2} \biggr) &\leq \frac{1}{2(mb-ma)} \int_{ma}^{mb}f(x)\,dx +\frac{m}{2(b-a)} \int_{a}^{b}f(x)\,dx \\ & \leq \frac{m}{2} \bigl( f(a)+f(b) \bigr). \end{aligned}$$

### Remark 3.3

If we take $m=1$ in (31) of Corollary (3.2)(2), then we get (2).

### Theorem 3.4

*Let* $\alpha >0$ *and* $\rho >0$. *Let* $f:[a^{\rho },b^{\rho }]\subset \mathbb{R}_{+}\rightarrow \mathbb{R}$ *be a positive function with* $0\leq a< b$ *and* $f\in X^{p}_{c}(a^{\rho },b^{\rho })$. *If* *f* *is also an* *m*-*convex function on* $[a^{\rho },b^{\rho }]$. *Let* $F(x^{\rho },y ^{\rho })_{t^{\rho }}:[0,1]\rightarrow \mathbb{R}$ *be defined as*

$$ F\bigl(x^{\rho },y^{\rho }\bigr)_{t^{\rho }}=\frac{1}{2} \bigl[ f\bigl(t^{\rho }x^{ \rho }+m^{\rho } \bigl(1-t^{\rho }\bigr)y^{\rho }\bigr) +f\bigl(\bigl(1-t^{\rho } \bigr)x^{\rho }+m ^{\rho }t^{\rho }y^{\rho }\bigr) \bigr] . $$

*Then we have*

32 $$\begin{aligned} &\frac{1}{(b^{\rho }-a^{\rho })^{\alpha }} \int_{a}^{b}\bigl(b^{\rho }-u ^{\rho } \bigr)^{\alpha -1}u^{\rho -1} F \biggl( u^{\rho },\frac{a^{\rho }+b ^{\rho }}{2} \biggr) _{ ( \frac{b^{\rho }-u^{\rho }}{b^{\rho }-a^{ \rho }} ) }\,du \\ &\quad \leq \frac{\rho^{\alpha -1}\Gamma (\alpha)}{2(b^{\rho }-a^{\rho })^{ \alpha }} {}^{\rho }I_{a+}^{\alpha }f \bigl(b^{\rho }\bigr)+\frac{m}{2\rho \alpha }f \biggl( \frac{a^{\rho }+b^{\rho }}{2} \biggr). \end{aligned}$$

### Proof

Since *f* is an *m*-convex function, we have

$$\begin{aligned} F\bigl(x^{\rho },y^{\rho } \bigr)_{t^{\rho }} &\leq \frac{1}{2} \bigl[ t^{\rho }f\bigl(x ^{\rho }\bigr)+m^{\rho }\bigl(1-t^{\rho }\bigr)f \bigl(y^{\rho }\bigr) +\bigl(1-t^{\rho }\bigr)f\bigl(x^{ \rho } \bigr)+m^{\rho }t^{\rho }f\bigl(y^{\rho }\bigr) \bigr] \\ & =\frac{1}{2} \bigl[ f\bigl(x^{\rho }\bigr)+m^{\rho }f \bigl(y^{\rho }\bigr) \bigr] , \end{aligned}$$

and also

$$ F \biggl( x^{\rho },\frac{a^{\rho }+b^{\rho }}{2} \biggr) _{t^{\rho }} \leq \frac{1}{2} \biggl[ f\bigl(x^{\rho }\bigr)+m^{\rho }f \biggl( \frac{a^{\rho }+b ^{\rho }}{2} \biggr) \biggr] . $$

Take $x^{\rho }=t^{\rho }a^{\rho }+(1-t^{\rho })b^{\rho }$, we have

33 $$ F \biggl( t^{\rho }a^{\rho }+\bigl(1-t^{\rho } \bigr)b^{\rho },\frac{a^{\rho }+b ^{\rho }}{2} \biggr) _{t^{\rho }}\leq \frac{1}{2} \biggl[ f\bigl(t^{\rho }a^{ \rho }+ \bigl(1-t^{\rho }\bigr)b^{\rho }\bigr)+m^{\rho }f \biggl( \frac{a^{\rho }+b^{ \rho }}{2} \biggr) \biggr] . $$

Multiplying both sides of (33) by $t^{\alpha \rho -1}$, $\alpha >0$ and then integrating the resulting inequality with respect to *t* over $[0,1]$, we obtain

34 $$\begin{aligned} & \int_{0}^{1}t^{\alpha \rho -1}F \biggl( t^{\rho }a^{\rho }+\bigl(1-t^{\rho }\bigr)b ^{\rho }, \frac{a^{\rho }+b^{\rho }}{2} \biggr) _{t^{\rho }}\,dt \\ &\quad \leq \frac{1}{2} \int_{0}^{1}t^{\alpha \rho -1} \biggl[ f \bigl(t^{\rho }a ^{\rho }+\bigl(1-t^{\rho } \bigr)b^{\rho }\bigr) +m^{\rho }f \biggl( \frac{a^{\rho }+b ^{\rho }}{2} \biggr) \biggr]\,dt. \end{aligned}$$

Then, by the change of variable $u^{\rho }=t^{\rho }a^{\rho }+(1-t ^{\rho })b^{\rho }$, we get the desired inequality (32). □

### Remark 3.5

By taking $\rho =1$ in (32) of Theorem 3.4, we get Theorem 6 in [22].

## Applications to special means

In this section, we consider some applications to our results. Here we consider the following means:

1. The arithmetic mean:
  $$ A(a,b)=\frac{a+b}{2}; \quad a,b\in \mathbb{R}. $$
2. The logarithmic mean:
  $$ L(a,b)=\frac{\ln \vert b \vert -\ln \vert a \vert }{b-a}; \quad a,b\in \mathbb{R}, \vert a \vert \neq \vert b \vert , a,b\neq 0. $$
3. The generalized log mean:
  $$ L_{n}(a,b)= \biggl[ \frac{b^{n+1}-a^{n+1}}{(n+1)(b-a)} \biggr] ^{1/n} ;\quad a,b\in \mathbb{R}, n\in \mathbb{Z}\setminus \{-1,0\}, a,b\neq 0. $$

### Proposition 4.1

*Let* $a,b\in \mathbb{R}$, $a< b$, $0\notin [a,b]$, *and* $n\in \mathbb{Z}$, $|n|\geq 2$, *then*

35 $$ \biggl\vert A\bigl(a^{n},b^{n}\bigr)- \frac{b-a}{b+a}L_{n}^{n}(a,b) \biggr\vert \leq \frac{ \vert n \vert (b-a)}{2}A \bigl( \vert a \vert ^{n-1}, \vert b \vert ^{n-1} \bigr). $$

### Proof

By taking $f(x)=x^{n}$ in Corollary 2.4(3), we get the required result. □

### Proposition 4.2

*Let* $a,b\in \mathbb{R}$, $a< b$, $0\notin [a,b]$, *and* $n\in \mathbb{Z}$, $|n|\geq 2$. *Then*, *for* $q\geq 1$, *we have*

36 $$ \biggl\vert A\bigl(a^{n},b^{n}\bigr)- \frac{b-a}{b+a}L_{n}^{n}(a,b) \biggr\vert \leq \frac{ \vert n \vert (b-a)}{2^{2-1/q}}A^{1/q} \bigl( \vert a \vert ^{q(n-1)}, \vert b \vert ^{q(n-1)} \bigr). $$

### Proof

By taking $f(x)=x^{n}$ in Corollary 2.8(3), we get the required result. □

### Proposition 4.3

*Let* $a,b\in \mathbb{R}$, $a< b$, $0\notin [a,b]$, *and* $n\in \mathbb{Z}$, $|n|\geq 2$. *Then*, *for* $q\geq 1$, *we have*

37 $$ \biggl\vert A\bigl(a^{n},b^{n}\bigr)- \frac{b-a}{b+a}L_{n}^{n}(a,b) \biggr\vert \leq \frac{ \vert n \vert (b-a)}{2}A^{1/q} \bigl( \vert a \vert ^{q(n-1)}, \vert b \vert ^{q(n-1)} \bigr). $$

### Proof

By taking $f(x)=x^{n}$ in Corollary 2.10(3), we get the required result. □

### Proposition 4.4

*Let* $a,b\in \mathbb{R}$, $a< b$, $0\notin [a,b]$, *and* $n\in \mathbb{Z}$, $m\in [0,1]$, *then we have*

38 $$ f\bigl(mA(a,b)\bigr)\leq \frac{1}{2}L_{n}^{n}(ma,mb)+ \frac{m}{2}L_{n}^{n}(a,b) \leq mA \bigl(a^{n},b^{n}\bigr). $$

### Proof

By taking $f(x)=x^{n}$ in Corollary 3.2(2), we get the required result. □

### Proposition 4.5

*Let* $a,b\in \mathbb{R}$, $a< b$, $0\notin [a,b]$, *then*

39 $$ \biggl\vert A\bigl(a^{-1},b^{-1}\bigr)- \frac{b-a}{b+a}L(a,b) \biggr\vert \leq \frac{b-a}{2}A \bigl( \vert a \vert ^{-2}, \vert b \vert ^{-2} \bigr). $$

### Proof

By taking $f(x)=\frac{1}{x}$ in Corollary 2.4(3), we get the required result. □

### Proposition 4.6

*Let* $a,b\in \mathbb{R}$, $a< b$, $0\notin [a,b]$. *Then*, *for* $q\geq 1$, *we have*

40 $$ \biggl\vert A\bigl(a^{-1},b^{-1}\bigr)- \frac{b-a}{b+a}L(a,b) \biggr\vert \leq \frac{b-a}{2^{2-1/q}}A^{1/q} \bigl( \vert a \vert ^{-2q}, \vert b \vert ^{-2q} \bigr). $$

### Proof

By taking $f(x)=\frac{1}{x}$ in Corollary 2.8(3), we get the required result. □

### Proposition 4.7

*Let* $a,b\in \mathbb{R}$, $a< b$, $0\notin [a,b]$. *Then*, *for* $q\geq 1$, *we have*

41 $$ \biggl\vert A\bigl(a^{-1},b^{-1}\bigr)- \frac{b-a}{b+a}L(a,b) \biggr\vert \leq \frac{b-a}{2}A^{1/q} \bigl( \vert a \vert ^{-2q}, \vert b \vert ^{-2q} \bigr). $$

### Proof

By taking $f(x)=\frac{1}{x}$ in Corollary 2.10(3), we get the required result. □

### Proposition 4.8

*Let* $a,b\in \mathbb{R}$, $a< b$, $0\notin [a,b]$, *and* $m\in [0,1]$, *then we have*

42 $$ f\bigl(mA\bigl(a^{-1},b^{-1}\bigr)\bigr)\leq \frac{1}{2}L(ma,mb)+\frac{m}{2}L(a,b)\leq mA\bigl(a ^{-1},b^{-1}\bigr). $$

### Proof

By taking $f(x)=\frac{1}{x}$ in Corollary 3.2(2), we get the required result. □

## Conclusion

In Sect. 2, some Hermite–Hadamard type inequalities for *s*-convex functions in a generalized fractional form were obtained. In Corollaries 2.4, 2.8, and 2.10, we obtained some new results related to *s*-convex functions, convex functions via Riemann–Liouville fractional integrals and via classical integrals. In Sect. 3, we established a Hermite–Hadamard type inequality for *m*-convex functions in generalized fractional integrals. In Corollary 3.2, a new Hermite–Hadamard type inequality for *m*-convex functions via Riemann–Liouville fractional integrals and via classical integrals was proved.
